# Tailoring the Scattering Response of Optical Nanocircuits Using Modular Assembly

**DOI:** 10.3390/nano12172962

**Published:** 2022-08-27

**Authors:** Sajid Farooq, Shareen Shafique, Zishan Ahsan, Olavo Cardozo, Faiz Wali

**Affiliations:** 1 Center for Lasers and Applications, Instituto de Pesquisas Energéticas e Nucleares, IPEN-CNEN Av. Prof. Lineu Prestes, 2242-Cidade Universitária, São Paulo 05508-000, Brazil; 2 Laboratory of Biomedical Optics and Imaging, Federal University of Pernambuco, Recife 50000-000, Brazil; 3 State Key Laboratory for Manufacturing Systems Engineering, Xi’an Jiaoting University, Xi’an 710049, China; 4 Key Laboratory of Green Fabrication and Surface Technology of Advanced Metal Materials, Ministry of Education, School of Materials Science and Engineering, Anhui University of Technology, Maanshan 243000, China; 5 Phornano Holding GmbH, 2100 Korneuburg, Austria; 6 Key Laboratory of Optoelectronic Devices and Systems of Ministry of Education and Guangdong Province, College of Physics and Optoelectronic Engineering, Shenzhen University, Shenzhen 518060, China

**Keywords:** nanocircuits, plasmonics, lumped elements

## Abstract

Owing to the localized plasmon resonance of an ensemble of interacting plasmonic nanoparticles (NPs), there has been a tremendous drive to conceptualize complex optical nanocircuits with versatile functionalities. In comparison to modern research, there is still not a sufficient level of sophistication to treat the nanostructures as lumped circuits that can be adjusted into complex systems on the basis of a metatronic touchstone. Here, we present the design, assembly, and characterization of single relatively complex photonic nanocircuits by accurately positioning several metallic and dielectric nanoparticles acting as modular lumped elements. In this research, Au NPs along with silica NPs were used to compare the proficiency and precision of our lumped circuit model analytically. On increasing the size of an individual Au NP, the spectral peak resonance not only modifies but also causes more scattering efficiency which increases the fringe capacitance linearly and decreases the nanoinductance of lumped circuit element. The NPs-based assembly induced the required spectral resonance ascribed by simple circuit methods and are depicted to be actively reconfigurable by tuning the direction or polarization of input signals. Our work demonstrates a vital step toward developing the modern modular designing tools of complex electronic circuits into nanophotonic-related applications.

## 1. Introduction

The concept of complex circuits with versatile functionalities as being composed of the appropriate elements of basic ‘lumped’ circuits, e.g., resistors, capacitors and inductors, has been a key element enabling the microelectronic technology advances [1,2]. In modeling, the “lumpedness” of circuit elements is an important assumption, allowing simplification and effective modularization of each element’s function. In contrast, modern nanoscale optical devices, due to a lack of modularization of their response in terms of basic building blocks, lag behind in terms of a similar level of sophistication. This modularization procedure, in electronics, leads to the design of complex nanocircuits by assembling individual and lumped circuit elements. A plethora of literature related to modularization has already been manipulated in metasurfaces [3], nanoantenna designs [4] and computational devices [5], optical metatronic circuits [6], opticla waveguides [7], as well as in optical nanofilters [1,8]. Over the last few decades, a variety of plasmonic nanodevices have been designed to localize light under the diffraction limit, create near-field enhancement of electromagnetic, and emphasize the mechanical effects of nano-circuits and their working in the quantum realm [9]. The optical properties of metallic nanoparticles (NPs) have been a subject of substantial experimental and theoretical interest [1,10]. In optical frequencies, certain noble metals such as Ag and Au behave as plasmonic materials, the plasma frequency is in the visible or ultraviolet (UV) regimes, as their permittivity has a negative real part [11]. As a result, the interaction of optical signals with plasmonic metals gives rise to surface plasmon resonances [12]. For instance, the absorption destructive and constructive features were realized in metallic nano-antennas through in-plane coherent control of plasmon resonances, determining by the distribution rule of electrical field components of plasmonic antennas [13].

By exploiting the optical properties of metamaterials and modularizing the optical response of these plasmonic metals (Au, Ag), they may play the role of “lumped” nanocircuit elements such as nanoinductors, nanocapacitors, and nanoresistors, analogous to microelectronics connected in a complex circuit [14]. The use of optical nanocircuit elements and modeling composite nanostructures is based on plasmonic NPs clusters [15,16] and single-layer or multiple-layer complicated metamaterial structures [3,5,17]. Moreover, the plasmonic nanocircuitry features such as guiding, routing and processing of aforementioned nanocircuits are based on enhancing the plasmon propagation length and improving the coupling efficiency [18].

The intrinsic optical impedance of NPs according to optical nanocircuit concepts [14,19], may be tailored by their material composition and geometry. That said, impedance is defined as the ratio of the local potential difference V = **|E|**$l_{d}$ and the displacement current flux $I_{d}$ = −iω|E|S through the NP [19], where **E** is the local electric field vector, $l_{d}$ is the length of NP along the electric field, ϵ is the particle’s dielectric constant, S is its transverse cross-section and ω is operational frequency [14]. It states that a dielectric NP with Re(ϵ)>0 behaves as a nanocapacitor, while a metallic NP with Re(ϵ)<0 acts as a nanoinductor. Further, ohmic loss in the material is responsible for resistance and behaves as a nanoresistor.

A general method to describe the optical response of plasmonic nanostructures is to bridge the gap between full-field electromagnetic calculations and equivalent lumped circuit elements. The equivalent circuit approach provided a useful framework connecting the exact numerical values of all the lumped elements accurately, and reproduces the behavior of complex plasmonic circuits (Fano resonances, retardation effects, and polarization coupling) [20]. The optical circuits elements, moreover, could be employed to systematically resolve the impedance-matching problems of plasmonic and photonic components, which are commonly mismatched with respect to each other as well as in free space, partially due to their nanometer size. As suitably designed and fabricated optical nanocircuits are proposed to match and tune the input impedance of nanoantenna [4]. Therefore, it is important to emphasize the appropriate assembly design of nanocircuits to explore optical nanocircuits as well as for matching purposes. Thus, the reliable modular assembly of NPs clusters and consistent confirmation of the underlying electromagnetic modes hinders complex circuits designing; also, translation of such particles clusters into broad meta-material application with extraordinary light guiding behavior still requires us to gauge the performance of metamolecule assembly while maintaining an exotic level of complexity of particle clusters with numerical manipulation for practical realization.

In this paper, we present the quantitative derivation of the equivalent circuit elements based on the electromagnetic field that can solve Maxwell’s equations using the computational simulation method. Commercially available numerical techniques such as COMSOL Multiphysics can provide the response of a plasmon-based nanostructure with great accuracy. Transmission electron microscope (TEM) was used to characterize the NPs for optical spectroscopic analysis. The TEM images of the scattered Au NPs show that our specimens are spherical with an average radius of 50 nm. The NPs-based assembly induces the required spectral resonance ascribed by simple circuit methods, which depicts efficient reconfigurability by tuning the direction or polarization of input signals. The absorbance spectrum exhibits spectral resonance at 550 nm, which is in good agreement experimentally as well as theoretically.

## 2. Materials and Methods

The lumped element model in electronics is a simple description of a distinctly divided physical system based on a topology (the study of geometric properties and spatial relations unaffected by the continuous change in shape or size) consisting of isolated entities. The smaller dimensions of the circuit elements contrasted to the operating wavelength justified the well-established estimation of lumped element model. The model under study is discussed briefly in the following sections.

### 2.1. Numerical Simulations Modeling

**Figure 1:**
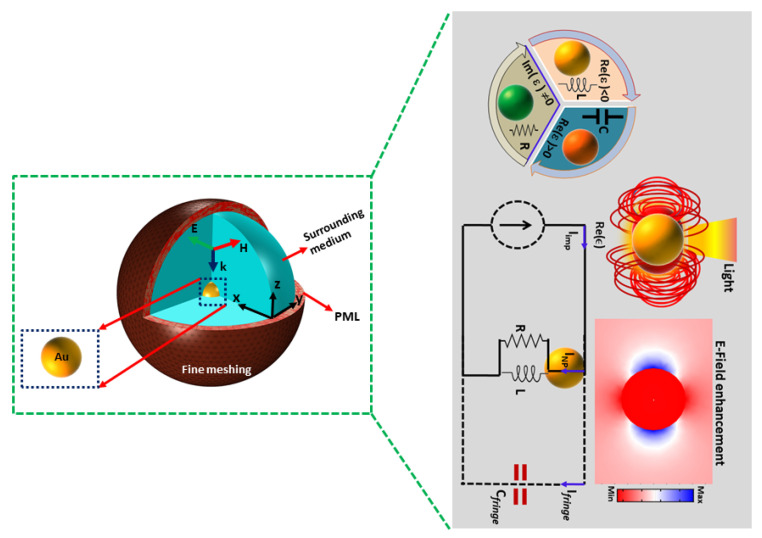
A 3D Schematic diagram for numerical simulation analysis. The Au NP is immersed in surrounding medium (*n* = 1) at the center of spherical domain and incident field is illuminated in the opposite direction of Z-axis of the domain.The whole computational domain is equivalent to nanocircuit for single Au NP.

Computational simulations were used to quantitatively explain the equivalent circuit elements, render the interaction of light with the Au nanosphere, which leads to the understanding of plasmon-based nanostructure with great accuracy. To evaluate the scattering cross-section ($\sigma_{sca}$) and localized field around the Au sphere as a result of interaction of electromagnetic radiation with nanosphere surface, the Finite Element Method (FEM) in frequency module in COMSOL Multiphysics is implemented. In FEM simulation, the nanoparticle surface was divided into small tetrahedral mesh elements with ‘finer’ size. Moreover, anisotropic perfectly matched layer (PML) around the nanosphere was used to avoid any reflection artifacts during the simulation, as shown in Figure 1. The background incident field was set to 1 Vm${}^{-1}$. The data of the metal complex (real and imaginary) dielectric function for Au was obtained from the literature [21]. To investigate the optical scattering cross-section ($\sigma_{sca}$), we used the solutions of Maxwell’s equations. Therefore, $\sigma_{sca}$ of the nanoparticle is given as [22]:

(1) $$\sigma_{sca}=\frac{1}{I_{0}}\int \int \left(n.S_{sca}\right)dS$$

where ***n*** is the normal from the center of Au NP, *S${}_{sca}$* is the scattered intensity vector over the surface area ‘*S*’ of the computational domain.

### 2.2. Circuit Theory

**Figure 2:**
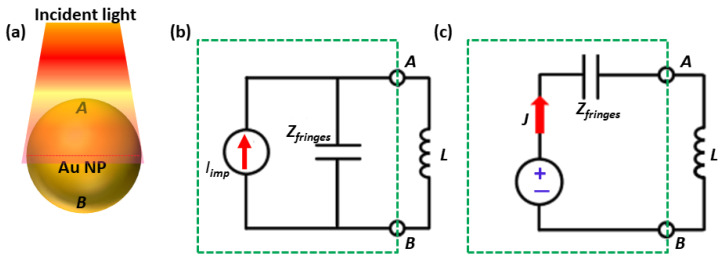
Plasmonic NP-based nanocircuit models. (**a**) Au NP with two terminals A and B as defined on the upper and lower hemispherical surfaces of the NP, (**b**) Norton and (**c**) Thevenin equivalent nanocircuits excited under the illumination of uniform field.

Our circuit model anticipates the scattering spectra showing quantitative dependability with the measured and simulated spectra for both S-circuits as well as for P-circuits. With this consistency, we predicted that a circuit model can be made more complex by just introducing a metal NP under the previous nano circuit design. The distribution of electrical displacement vectors reinforces circuit models even in this more complex design, which is actually nothing but a fourth order nano-filter. In S-circuit, an additional nanoinductor shares the same applied potential with the nanocapacitor thus in parallel connection, i.e., series circuit. On the other hand, in P-circuit that nanoinductor makes parallel combination with the third order lumped nano-filter. Moreover, the nanoinductor is connected in exactly the same way as in an electronic circuit to control the spectral response quantitatively. As compared to the equivalent circuit models for photonic structures previously developed [23,24], the nanocircuit paragon introduced by our numerical analysis is appreciably more substantial. We transform the original circuit in ref. [19] to its Thevenin equivalent as reported by Shi et al. [1] for relating our measurements to the circuit model. At resonance, the source delivers the current [25] at maximum as shown in Figure 2, where the scattering peak is associated with the reported experimental analysis. In accordance with the physical outlook, the polarization current is in actuality the current flowing in the circuit for sustaining the scattered fields. This issue can be better estimated by introducing two terminals, A and B, on the upper and lower hemispherical surfaces of the nanosphere (Figure 2a), splitting the impedance of the circuit element (the inductance *L* in this example) from the rest. One can see, in Figure 2b, that part of the terminals, consisting of the current source and the shunt fringe capacitance, can be replaced by its Thevenin equivalent circuit (Figure 2c), comprised of an equivalent voltage source $V_{th}$ and an equivalent impedance $Z_{th}$ in series combination with each other. Specifically, when the circuit is open the voltage obtained at the terminals A and B are regarded as the equivalent voltage. For a spherical particle of radius “R” and permittivity “ϵ”, the impressed current is $I_{imp}$ = −i$\omega \left(\epsilon -\epsilon_{0}\right)\pi R^{2}|E_{0}|$ where $\left|E_{0}\right|$ is the uniformly assumed incident electric field, and the capacitive impedance corresponding to the fringe field is $Z_{fringes}$ = (−i$\omega \epsilon_{0}{2\pi R)}^{-1}$. Thus, the equivalent voltage source is given as:

(2) $$\;V_{th}=I_{imp}\times Z_{fringes}=-\left(\epsilon_{r}-1\right)R\left|E_{0}\right|$$

Here, the relative permittivity is $\epsilon_{r}$, the current source acts as an open-circuit, and the impedance at the terminals *A* and *B* is regarded as the equivalent Thevenin impedance: that is why $Z_{th}$ = Z${}_{fringes}$. The final Thevenin equivalent circuit accompanied by the same nano-element impedance is consistent with the supposed circuit model. From the viewpoint of the nanocircuit, there are two equivalent representations due to the linearity of the electromagnetic problem, therefore, we can opt for the circuit model as the best choice for our measurements. The origination of this nanocircuit model is consequential to satisfactorily specifying the response of optical nanocircuits in the far-field.

### 2.3. Materials Analysis

Gold nanospheres colloidal samples with polyvinylpyrrolidone (PVP) stabilizer were obtained from nanoComposix. The samples absorbance spectrum, from 300 to 1100 nm, were obtained using an Ocean Optics spectrophotometer (HR + 4000), with a 1 nm resolution. For the spectroscopic analyses, the samples were placed in quartz cuvettes of 1 cm width. Fiber bundles were employed to shine light from a Halogen–Deuterium light source to the sample and to send light from sample to the spectrophotometer.

**Figure 3:**
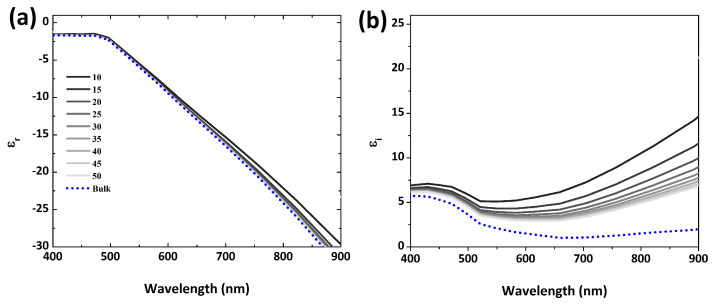
The dielectric function of gold nanoparticles (**a**) real part and (**b**) imaginary part of permittivity. The radius of particle is tuned from 10 nm to 50 nm with a step of 5 nm.

The proficiency and incompetence of a dielectric material for numerical simulations is determined by its dielectric constant, which indicates that how much electrical energy can be stored and lost by the material in the form of heat energy. The permittivity associated with the material or the corresponding medium becomes a complex quantity under the influence of an alternating current provided by the modeled LC circuit, thus, comprises of a real part as well as imaginary part. The real part $\epsilon_{r}$ refers to the degree of polarization (dielectric gain) and the imaginary part $\epsilon_{i}$ indicates dielectric losses (absorbed energy), that is why it is always positive. The appropriate realistic model to treat dielectric function of metals is free-electron or Drude model [11].

(3) $$\epsilon_{\omega }=\epsilon_{int}\left(\omega \right)+\frac{\omega_{p}^{2}}{\omega (\omega +i\gamma )}$$

where $\epsilon_{int}$ indicates the interband transitions, γ is phenomenological scattering parameter and $\omega_{p}$ represents plasmon frequency. By studying Drude model, the anticipation of γ relies on intrinsic characteristics of the material and from interface scattering by subwavelength NPs. The equation associates for scattering parameter is $\gamma =\gamma_{bulk}+\gamma_{sca}$. For example, the Au bulk damping constant ($\gamma_{bulk}$) and Fermi velocity ($V_{f}$) are $\gamma_{bulk}$ = 15 ${\left(fs\right)}^{-1}$ and $V_{f}$ = 1.4×10${}^{6}$ ms${}^{-1}$, respectively. For small noble NPs, there are plenty of electrons on the surface that cause electron-interface scattering when the effective electron path length ($L_{eff}$) is larger than to the NP itself. The scattering parameter ($\gamma_{sca}$) is achieved via ($\gamma_{sca}$) = $\frac{AV_{f}}{L_{eff}}$ where *A* is termed as scattering efficiency. The $L_{eff}$ for convex shapes NPs such as for spheres, rods, cubes etc., is obtained as $L_{eff}$ = $\frac{4V}{S}$ where *S* represents the surface area and *V* is the volume. The dielectric function is frequency dependent on the spontaneous polarization and the dielectric constant decreases as the NP size increases [26], which is depicted in Figure 3. It can be seen that reducing the $L_{eff}$ may tailor for both real and imaginary parts of gold dielectric function. The smaller negativity of $\epsilon_{r}$ depicts lower polarizability of Au NPs (Figure 3a), while increasing the magnitude of $\epsilon_{i}$ indicates the growing loss of the Au NP as shown in Figure 3b, respectively. Nanostructures and bulk manifest different material properties due to divergent surface area over volume ratio [25]. Thus, the small size NPs exhibit a smoother behavior as compared to bulky materials above ≅525 nm. The mere scaling of plasmonic devices introduced a new methodology known as “equivalent nano circuit (EN) theory” [27]. The initiative was taken from a basic LC optical nanocircuit based on a single Au nanosphere with radius 30 nm excited by a monochromatic field. To gain insights, lumped circuit elements are far more useful. They provide an emblematic illustration of the plasmonic system and optimize the application-driven design. The scattering efficiency obtained from NP can be precisely explained by a simple nanocircuit model in which both capacitor as well as inductor are connected in series.

## 3. Results

**Figure 4:**
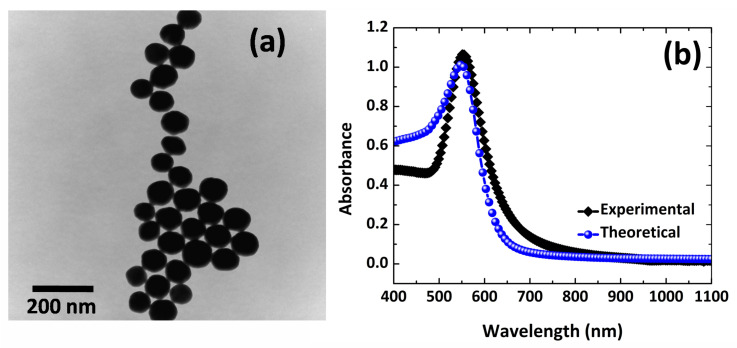
(**a**) TEM image of Au NPs and scale is 200 nm. (**b**) Optical absorbance of Au NPs in colloid and comparison numerical study using FEM computational modeling and experimental analysis.

In our analysis, we initially used Au NPs to compare our model accuracy with the experimental one. As depicted in Figure 4 , the optical spectroscopic analysis and TEM were employed for the NPs characterization. TEM image of dispersed Au NPs, presents a spherical shape and 50 nm average radius of our samples (Figure 4a). Optical spectroscopic analysis was used to explore the properties of Au NP, as shown in Figure 4b. One can see in Figure 4b that absorbance spectra obtains experimentally and theoretically spectral resonance at 550 nm. The plasmonic properties (i.e., absorption, emission, extinction, scattering etc.) of nanostructures depend on aspect ratio, geometry and refractive index of the surrounding media. For instance, in the case of 1-D nanomaterials (nanorods and nanowires), the change in aspect ratio is accompanied by change in absorption in different regions [28]. Figure 4b shows the absorption spectrum for colloidal gold nanoparticles displaying a good agreement between experimental and theoretical results, such that the optimal absorption (peak) obtained at ≅550 nm consistent with the experimental result in visible region. The absorption cross-section spectra obtained by the simulation method, identical the values obtained experimentally, validating our FEM model. These results confirm the great accuracy of our FEM simulations model to further analyze our model for nanocircuits analysis.

**Figure 5:**
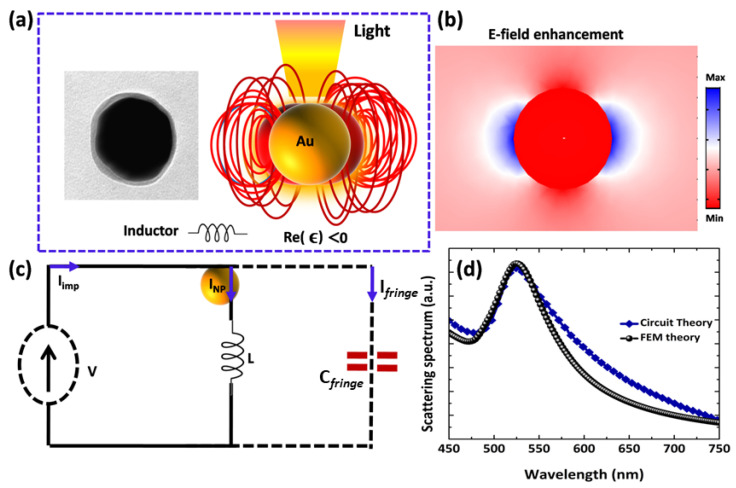
(**a**) TEM image and a schematic of an individual Au sphere inserted in surrounding medium. Simulated fringe dipolar field lines around the Au NP are negative (**b**) with optical scattered localized field enhancement. (**c**) A plasmonic nanoinductor based on Au NP realizing a lumped LC circuit and obtained from Thevenin-equivalent model. (**d**) Comparison between the scattering spectrum obtained with FEM based simulations (black line) and the response calculated by nanocircuit theory (blue line; amplitude of the current flowing in the circuit (**c**)).

To explore basic optical LC circuit, we use single Au NP of R = 50 nm, under the illumination of a uniform field, as expounded in Figure 5a. Figure 5b represents the scattered fields supported by the polarization currents induced in the NPs. The polarization currents have a direct relation with displacement current (as shown by blue arrow in Figure 5c) passing through the circuit that is driven by a Thevenin voltage generator (TVG). The current amplitude (the blue curve in Figure 5d) depends on the frequency using circuit theory and compared with the scattering spectra of finite element method simulations as reported by the black curve in Figure 5d. An individual Au NP (ϵ<0), in this case, is represented as basically a nanoinductor with optical impedance $Z_{NP}={\left(-i\omega \pi \epsilon R\right)}^{-1}$ where ϵ is the complex dielectric function of Au with capacitor impedance $Z_{fringes}$ and $G_{NP}=\pi \omega R\epsilon_{i}$. For $\epsilon_{r}>0$, in case of dielectric material such as SiO${}_{2}$ (R = 50 nm), the capacitance of the particle can be calculated as $C_{NP}$= $\pi R\epsilon_{d}$ where $\epsilon_{d}$ is dielectric function of SiO${}_{2}$. Moreover, the fringe capacitance of LC circuits is obtained as $C_{fringes}=2\pi R\epsilon_{0}$ where $\epsilon_{0}$ is free space permittivity. The optical circuit is operated by a TVG related to the applied field. For instance, the geometrical and material features of a Au NP (R = 50 nm) induce a nanoinductance $L_{NP}$ = 11.8 femtoH, $G_{NP}$ = 11.1 mS and $C_{fringes}$ = 2.75 attoF at $\lambda_{res}$ = 536 nm. One can notice that the proposed circuit is different from the original as reported in ref. [19] but similar to recent research [1]. The frequency reliant on the current amplitude (the blue curve in Figure 5d) matches well with the scattering signals obtained numerical (black curve), which are obtained from full-wave simulations (Figure 5d). It is quite clear that the proposed nanocircuit model, despite its simplicity, encapsulates not only the plasmonic resonance but also the resonance based quality factor and frequency dispersion of calculated spectrum.

**Figure 6:**
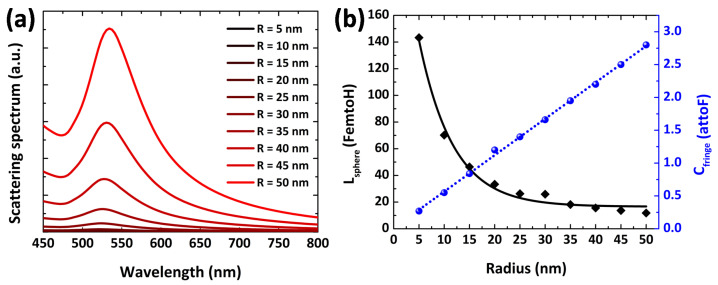
(**a**) The optical spectrum of Au nanospheres on tuning their radius from 5 nm to 50 nm in air as a surrounding medium. (**b**) The inductance and capacitance as a function of radius of Au NP evaluating using circuit theory (black squares) and Mie theory (blue diamond).

In Figure 6, the optical scattering spectrum is obtained for Au NPs on tuning the size from 5 nm to 50 nm with a step size of 5 (radius of Au NP) to evaluate the particle size effect on nanocircuit performance. It is evident thatthe larger the size of Au NP, the higher the scattering peak (i.e., more light scattering is achieved) [29]. In Figure 6a, we can see that the wavelength peak shift and scattering cross-section enhance as the NP radius increases. On changing the radius of Au NPs from 5 nm to 50 nm, the resonance peaks shift from 519 nm to 536 nm. On the other hand, nanoinductance continuously decreases with respect to the increment of radius. Moreover, $C_{fringes}$ of the nanocircuit increases linearly as a function of particle size, as depicted in Figure 6b. These results evidence that Au NPs within quasi-static limit (R ≤ 20 nm) depict higher L${}_{NP}$ values instead of bigger nanoparticles where optical scattering is high, as depicted in Table 1. It is clear from Table 1 that there is an influence of particle size on nanocircuits’ characteristics (L${}_{NP}$ and G${}_{NP}$) based on a single NP.

**Figure 7:**
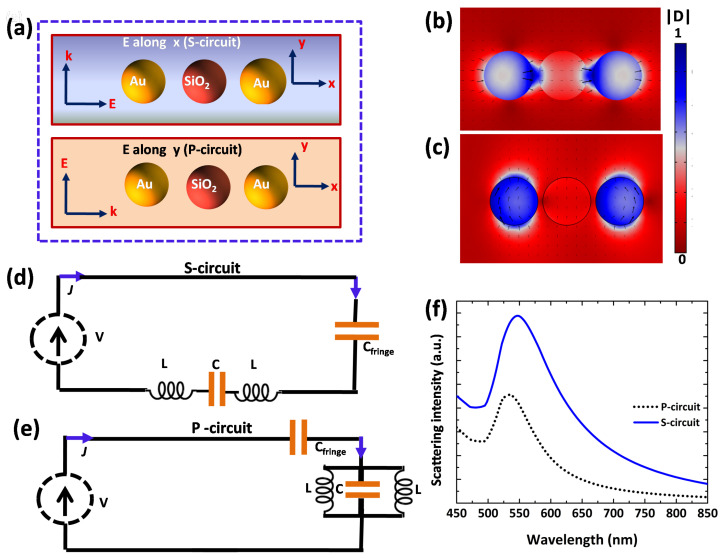
Second and third order lumped nanocircuits. (**a**) A schematic and geometry structure of a three NP trimmer composed of a silica nanosphere sandwiched between two Au nanospheres in air for perpendicular and parallel cases of incidence of light. (**b**) The simulated scattered displacement field measurements for s-polarized light exciting the nanocircuit with the E-field along x and localized field at the plasmonic resonance (547 nm). (**c**) The simulated scattered displacement field for p-polarized light exciting the nanocircuit with the E-field along y and response at the plasmonic resonance (532 nm). (**d**,**e**) Thevenin nanocircuit models of second order (d, series LC, S-circuit) and third order (i.e., CLC circuit, P-circuit) lumped nanofilters. (**f**) Corresponding Optical spectrum for S-circuit (solid blue line) and P-circuit (black dotted line).

Now we explore a three-particle nanocircuit in which silica nanosphere is sandwiched between two Au NPs of symmetrical size and shape, as presented in Figure 7a. The inter-particle distance is fixed, i.e., 10 nm, and particles possess the same radius. Different electric displacement field distributions are shown (Figure 7b,c) on the basis of which two circuits are derived, having distinguished connections among the circuit elements (Figure 7d,e), due to which different scattering spectra are obtained (Figure 7f). The robust hot spots are created when the s-polarized light is parallel to NPs array. Figure 7d shows an S-circuit with three nanocircuit elements in series combination, in which polarization light takes place either along the axis of the NPs array (S-circuit) or perpendicular to the NPs array (P-circuit). Figure 7f shows that a red shift in scattering resonance is obtained for S-circuit due to the increase in capacitive coupling within the NP’s array. The resonance wavelengths for S-circuit and P-circuit are 547 nm and 532 nm, respectively. The calculated fringe capacitance for S-circuit and P-circuit are, respectively, 8.4 aF and 0.9 aF.

Two distinguishable circuits are formed (different nanocircuit element connections) due to which different scattering spectra are obtained (Figure 7f). The peak shift in the scattering spectra is due to anisotropic properties of the nanocircuit elements. Figure 7d consists of a S-circuit with new nanocapacitor parallel to the previously analyzed third order nanofilter (three nanocircuit elements in series combination) in which s-polarization of incident light takes place. Conversely, Figure 7e comprises of a P-circuit with the same nanocapacitor connected in series to the previously designed third order nanofilter (three nanocircuit elements in parallel combination) in which p-polarization of incident light takes place.

**Figure 8:**
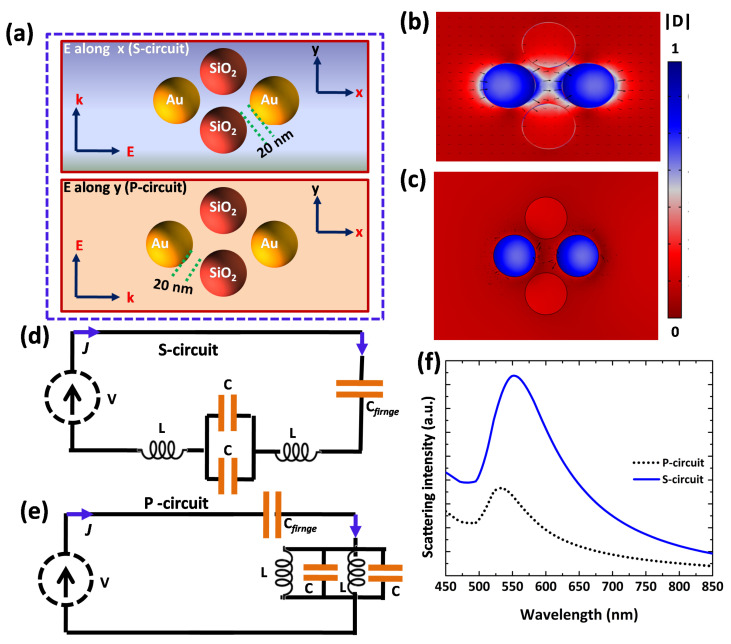
A fourth order lumped filter. (**a**) A schematic and geometry structure of a four NP complex nanocircuit composed of two silica nanospheres and two Au nanosphers for perpendicular and parallel cases of polarization. (**b**) The scattered displacement field calculated for s-polarized light shining the circuit with the incident field along x and localized field measured at the resonance (551 nm). (**c**) The simulated scattered displacement field for p-polarized light exciting the circuit with the E-field along y and response at the resonance (532 nm). (**d**,**e**) Thevenin nanocircuit models for S-circuit and P-circuit, respectively, representing lumped nanofilters. (**f**) The optical scattering spectrum for S-circuit (solid blue line) and P-circuit (black dotted line).

Now we elaborate a more complex nanocircuit (forth order nanofilter) in which an additional circuit element is considered, i.e., silica nanosphere is added below the previously described nanofilter, as shown in Figure 8a. The simulated vector displacement distributions are shown in (Figure 8b,c) and hot spots are generated at the nano-gaps when field is along *x*-axis. The interactions among nanoparticles depend on polarizationm as is evidently reported by Figure 8b, in which polarization parallel to the array, defining simulated vector displacement distribution at longer wavelength (551 nm) and Figure 8c in which polarization perpendicular/orthogonal to the array, defining simulated vector displacement distribution at shorter wavelength (532 nm). The fringe capacitance values for S-circuit and P-circut are, respectively, 5.6 aF and 0.7 aF.

## 4. Discussion

In this section, we would like to elaborate the main features of this study: the under probe nanocircuit model provides an unmatched degree of simplicity to tailor and optimize the optical response of nanophotonic structures. Remarkably, the impedance of each nanosphere, which is directly calculated using the relation $Z_{NP}={\left(-i\omega \pi \epsilon R\right)}^{-1}$, remains independent of the cluster geometry and surrounding medium in all the circuits, from isolated nanocircuit to the fourth order complex nanocircuit as discussed in Figure 8. More specifically, each nano-aggregate carries an inherent plasmonic impedance that makes them suitable candidates for simple and complex configurations and allowing one to modularize their response. The distance of nanostructures in the vicinity is expected to influence the overall response of the cluster. By considering the small gaps between neighboring particles, the flux of electric displacement vector is continuous (in case of the series) from one particle to the next one. On the contrary, in parallel connection, the electric field line integral across two neighboring nanostructures is the same. For small gaps, it is possible to neglect nonlocal effects. As the gaps increase, the associated impedance must be considered which may result in noticeable spectral shifts, but the overall nanoplasmonic circuit response is preserved. Briefly, without imposing the geometry and symmetry limitations, we demonstrated the plasmonic nanocircuit design and operation. The optical response of the lumped elements paved the way to design the complex photonic nanocircuits in different configurations. Moreover, in the study discussed above, we compared the response in the far-field limited by diffraction as explained in an experimental study for single Au NP by Shi et al. [1] and obtained good agreement between our full-wave simulation and the nanocircuit theory. In lieu of these findings, the plasmonic nanocircuits consisting of different materials open a paradigm for a variety of thought-provoking prototypical nanocircuits for parallelized optical signal processing at the nanoscale.

## 5. Conclusions

In a nutshell, the design and operation of a plasmonic nanocircuit are manifested without imposing geometry or symmetry constraints. Complex optical nanocircuits are made up of desired lumped elements, such as nanoinductors and nanocapacitors. We employed the use of gold nanoparticles in our analysis to make a comparison of the proficiency and precision of introduced lumped circuit model with experiments. Optical spectroscopic analysis was successfully employed to investigate the optical properties of Au nanoparticles (i.e., absorption, emission, extinction, scattering etc.). Despite the simplicity of the suggested nanocircuit model, it encompasses not only the plasmonic resonance but also the resonance-based quality factor and frequency distribution of the estimated spectrum. The TEM images of our dispersed gold nanoparticles show that they are spherical with an average radius of 50 nm. The optical response of the lumped elements preceded the complex photonic nanocircuits to be designed in various configurations. The response in the far-field constrained by diffraction in an individual NP case was measured and a good agreement between full-wave simulations and nanocircuit theory was found.

## Figures and Tables

**Table 1:** Effects of Au particles size on plasmonic resonance, nano-inductance and $G_{NP}$.

Radius	$\epsilon_{r}+i\epsilon_{i}$	$\lambda_{\mathrm{res}}$	$L_{\mathrm{NP}}$	$G_{\mathrm{NP}}$
		(nm)	(FemtoH)	(mS)
**5 nm**	−3.82 + i2.66	519	143.3	1.3
**10 nm**	−3.90 + i2.61	520	70.2	2.6
**15 nm**	−3.96 + i2.57	521	46.4	3.8
**20 nm**	−4.02 + i2.56	522	33.4	5.2
**25 nm**	−4.10 + i2.54	523	26.3	6.4
**30 nm**	−4.23 + i2.51	525	25.9	7.5
**35 nm**	−4.57 + i2.42	530	18.2	8.4
**40 nm**	−4.71 + i2.39	532	15.6	9.4
**45 nm**	−4.85 + i2.35	534	13.7	10.0
**50 nm**	−4.98 + i2.32	536	11.8	11.1

## Data Availability

The data that assists the findings of current research are available from the leading/first author, S.F., upon reasonable request.
